# Plasmacytoid Dendritic Cells Are Largely Dispensable for the Pathogenesis of Experimental Inflammatory Bowel Disease

**DOI:** 10.3389/fimmu.2018.02475

**Published:** 2018-10-25

**Authors:** Catherine M. Sawai, Lee Serpas, Antonio Galvao Neto, Geunhyo Jang, Ali Rashidfarrokhi, Roland Kolbeck, Miguel A. Sanjuan, Boris Reizis, Vanja Sisirak

**Affiliations:** ^1^Department of Pathology, New York University School of Medicine, New York, NY, United States; ^2^INSERM, ACTION Laboratory, University of Bordeaux, Bordeaux, France; ^3^Department of Respiratory, Inflammation and Autoimmunity, MedImmune LLC, Gaithersburg, MD, United States; ^4^Department of Medicine, New York University School of Medicine, New York, NY, United States; ^5^CNRS-UMR, Immunoconcept, Université de Bordeaux, Bordeaux, France

**Keywords:** Plasmacytoid dendritic cell (PDC), Interferon Type I, colitis, Inflammatory bowel disease (IBD), autoimmune disease

## Abstract

Inflammatory bowel disease (IBD) is a chronic inflammatory condition caused by an aberrant immune response to microbial components of the gastrointestinal tract. Plasmacytoid dendritic cells (pDCs) are innate immune cells specialized in the production of type I interferons and were recently implicated in the pathogenesis of autoimmune disorders such as lupus and scleroderma. While pDCs were shown to infiltrate intestinal mucosa of IBD patients and proposed to participate in intestinal inflammation, their net contribution to the disease remains unclear. We addressed this question by targeting the pDC-specific transcription factor TCF4 (E2-2) in experimental IBD caused by deficiency of Wiskott-Aldrich syndrome protein (WASP) or of interleukin-10 (IL-10). Monoallelic *Tcf4* deletion, which was previously shown to abrogate experimental lupus, did not affect autoimmunity manifestations or colitis in WASP-deficient animals. Furthermore, conditional biallelic *Tcf4* targeting resulted in a near-complete pDC ablation, yet had no effect on the development of colitis in IL-10-deficient mice. Our results suggest that, in contrast to other inflammatory and autoimmune diseases, pDCs do not play a major role in the pathogenesis of intestinal inflammation during IBD.

## Introduction

Dendritic cells (DCs) are major antigen-presenting cells that are essential for the initiation and regulation of immune responses. DCs are commonly subdivided into classical or conventional DCs (cDCs) and plasmacytoid DCs (pDCs). The pDCs are a distinct lineage whose function, phenotype, and core gene expression program are conserved across mammalian species (1, 2). They express a specific set of pathogen recognition receptors (PRR) including endosomal Toll-like receptor (TLR)-7 and TLR9 that recognize the nucleic acid ligands single stranded (ss)-RNA and unmethylated CpG-containing DNA, respectively (1, 2). In response to such stimuli pDCs rapidly produce copious amounts of type I interferon (IFNα/β, IFN-I), up to 1,000 times more than any other cell type (1, 2). IFN-I produced by pDCs is essential for inducing the expression of multiples genes with anti-viral properties and activates a broad range of immune cells subsequently providing anti-viral immunity (1, 2). In addition to their role in anti-viral immune responses, pDCs were shown to produce high amounts of IFN-I in response to self-nucleic acids and thus contribute to the development of inflammatory and autoimmune disorders (3–9). For instance, systemic lupus erythematous (SLE) patients (10, 11) have an accumulation of IFN-I-producing pDCs in the affected tissues, which likely contributes to the increased expression in IFN-I-inducible genes and overall “IFN-I signature” characteristic of SLE. In addition, recent studies using murine disease models that specifically lack pDCs have further confirmed their contribution to the pathogenesis of SLE (12, 13), systemic sclerosis (14), type I diabetes (15), and type II diabetes (16). These studies have provided a rationale for targeting pDCs as a therapeutic approach for such diseases, and antibodies that deplete pDCs in humans are currently being developed (17).

Within the small and large intestine pDCs are found primarily in the lamina propria (LP) and gut-associated lymphoid tissues (18). A distinct population of pDCs with reduced ability to produce IFN-I is also thought to populate Peyer's patches (19, 20). pDCs were previously proposed to contribute to B cell production of intestinal immunoglobulin A (IgA) (21). However, these results were recently challenged by the observation that pDC ablation had no major impact on intestinal IgA production *in vivo* (22). Through the production of IFN-I, intestinal pDCs were shown to participate in the clearance of enteropathogenic viruses such as rotavirus (23). Conversely, intestinal pDCs were described to mediate the tolerogenic effects of polysaccharide A (PSA), an immunomodulatory molecule of the gut commensal *Bacteroides fragilis* (24), and liver pDCs were implicated in the establishment of tolerance in response to ingested/oral antigens (25, 26). Thus, pDCs are clearly present in the gastrointestinal tract and appear to have context-dependent functions. Given the abundance of immunostimulatory microbial DNA/RNA in the intestine, pDCs may also contribute to the development of autoimmune and inflammatory conditions in the gastrointestinal tract.

Inflammatory bowel disease (IBD) comprises two major syndromes, Crohn's disease (CD) and ulcerative colitis (UC), which are progressive inflammatory conditions that affect the entire gastrointestinal tract and the colonic mucosa, respectively. IBD development is caused by dysregulated immune responses in genetically predisposed individuals to microbial components of the gastrointestinal tract (27). A role for pDCs in IBD has been suggested by the increase in their frequency and number in the inflamed intestinal mucosa of flaring IBD patients compared to healthy controls (28). In addition, pDCs from the peripheral blood (PB) of IBD patients generally display an activated phenotype defined by the increased expression of co-stimulatory molecules and the ability to spontaneously produce inflammatory cytokines such as tumor necrosis factor (TNF)-α and interleukin (IL)-6, but they also show impaired production of IFN-I in response to TLR7 and 9 stimulation (28). Another study documented increases in both activation of pDCs and IFN-I levels in Wiskott-Aldrich syndrome (WAS) patients who manifest a pleiotropic autoimmunity that includes colitis (29). Wiskott-Aldrich syndrome protein (WASP, gene symbol: *Was*)-deficient animals display a similar phenotype, including pDC hyperactivation and heightened IFN-I production. Furthermore, this study showed that the overall pathology including colitis in *Was*-deficient animals was ameliorated by IFN-I receptor (*Ifnar*) deficiency (29). While IFN-I clearly plays an important role in the development of colitis in *Was*-deficient animals, whether such IFN-I originates from pDCs and whether pDCs contribute at all to the overall pathology in this model was not tested. Finally, a recent study used genetic ablation of pDCs in an experimental model of colitis induced by dextran sodium sulfate (DSS) treatment and concluded that pDCs play a pathogenic role in IBD (30). However, in this model pDC ablation was achieved by diphtheria toxin (DT) treatment of Siglec H-DTR mice, which also depletes subsets of macrophages and DC progenitors, calling into question its specificity (31). Thus, the role of pDCs in the pathogenesis of IBD, if any, remains unclear and requires further study.

We have previously identified the E-protein transcription factor encoded by the gene *Tcf4* (E2-2) as a master regulator of pDC development in humans and mice (32). TCF4 is preferentially expressed in pDCs, and its deletion abolishes the development of pDCs but not of other immune cells including cDCs. Importantly, even monoallelic loss of *Tcf4* causes a specific defect in pDC function in mice and human patients. Indeed, *Tcf4*^+/−^ mice show aberrant pDC phenotype and impaired pDC-driven IFN-I response to the TLR9 agonist CpG-oligodeoxynucleotide (CpG-ODN) (32). Using *Tcf4* haplodeficiency for specific functional impairment of pDCs, we have demonstrated a crucial role for pDCs in two genetic models of experimental SLE (13). Furthermore, monoallelic or complete deletion of *Tcf4* in the DC lineage confirmed the deleterious function of pDCs in SLE (13) and showed a pathogenic role in autoimmune diabetes in NOD mice (15), respectively. Using a similar approach, here we explored the role of pDCs in two distinct models of IBD. We established *Tcf4* haplodeficiency in *Was*-deficient animals that are prone to systemic autoimmunity and that develop severe colitis with 100% penetrance. Additionally, we conditionally deleted *Tcf4* in DCs, which results in a specific deficiency in pDCs, in IBD-prone *Il10*-deficient mice (33). Although targeting of *Tcf4* in *Was-* or *Il10-*deficient mice altered and selectively depleted pDCs, respectively, we did not observe any further impact on the development of IBD in either model. Thus, our study using two distinct genetic models of the disease suggests that pDCs do not play a major role in genetically-induced IBD pathogenesis.

## Materials and methods

### Mice

All experiments were performed according to the investigator's protocol approved by the Institutional Animal Care and Use Committee of New York University Langone Medical Center. *Tcf4*^+/−^ (E2-2^+/−^) animals (34) were on pure 129SvEvTac (129Sv) background (>N12); all other animals were on pure C57BL/6J (B6) background (>N12). *Was*-deficient females (Strain 019458, The Jackson Laboratory) were crossed with *Tcf4*^+/−^ males to generate B6.129F1 *Was*^−/y^ *Tcf4*^+/−^ males or *Was*^−/y^ *Tcf4*^+/+^ littermates. Age-matched B6.129F1 males bred in the same colony were used as WT controls. For the conditional targeting of *Tcf4, Il10*-deficient animals (Strain 002251, The Jackson Laboratory) were crossed with the *Itgax* (CD11c)-Cre transgenic mice (35) floxed for *Tcf4* alleles (36) to generate *Il10*^−/−^ *Tcf4*^Fl/Fl^ mice with or without the *Itgax*-Cre transgene. Age-matched B6 males bred in the same colony were used as WT controls. No phenotypic differences have been observed between male and female *Il10*-deficient animals; thus, both male and female mice were included in the analysis.

### Macroscopic colitis assessment

*Was*-deficient *Tcf4*^+/+^ or *Tcf4*^+/−^ mice as well as their respective WT controls were euthanized at the age of 6 months. *Il10*^−/−^ *Tcf4*^*Fl*/*Fl*^ either positive or negative for the *Itgax*-Cre transgene together with their respective WT control were euthanized at 3 months. The colons were extracted, emptied of their content, photographed and measured for length. Samples from the colon were harvested and stored in TRIzol (Thermo Fisher) for RNA extraction. Animals were also followed prior to their euthanasia, and the occurrence of rectal prolapse was monitored on a weekly basis.

### Flow cytometry

Single cell suspensions were prepared from the spleen, lymph nodes and mesenteric lymph nodes by tissue digestion in the presence of collagenase D (1 mg/ml, Sigma Aldrich) and DNase1 (20 μg/ml, Sigma Aldrich) at 37°C for 30 min and filtration through a cell strainer 70 μm (Thermo Fisher). Cell suspensions were subjected to red blood cell lysis, washed, and stained with unconjugated and conjugated fluorescent antibodies listed in Table S1. Intracellular staining for Foxp3 and BCL6 were performed using the transcription factor staining buffer set (Thermo Fisher) according to the manufacturer's instructions. For optimal resolution of CXCR5 across CD4^+^ T cell subsets, staining with CXCR5 biotinylated antibody to identify CD4^+^ T follicular helper (Tfh) cells was performed for 1 h at room temperature, prior to conventional staining with other relevant markers. Samples were acquired on an Attune flow cytometer (Thermo Fisher) and analyzed using FlowJo software (Tree Star).

### Autoantibody measurement

Anti-dsDNA IgG concentration in the sera of animals was determined by ELISA as previously described (37) using calf thymus DNA as antigen. Anti-nuclear antibodies were detected by staining fixed HEp-2 cells (MBL Bion) with mouse serum (1:100 dilution), followed by PE-labeled goat anti–mouse IgG (Thermo-Fisher). Images were captured on a confocal fluorescent microscope (LSM 710 NLO) and processed by ZEN software (Carl Zeiss). For the profiling of IgG and IgM autoantibodies in the sera of mice we used a 98-plex autoantigen array provided by the UT Southwestern Genomics and Microarray Core facility. Briefly, samples were treated with DNase1, diluted 1/50 and incubated with the autoantigen array. Autoantibodies binding to antigens on the array were detected with Cy3 labeled anti-IgG and Cy5 labeled anti-IgM antibodies, and the arrays were scanned with a GenePix® 4400A Microarray Scanner. Images were analyzed using GenePix 7.0 software, and the averaged net fluorescent intensity (NFI) of each autoantigen was normalized to the internal control (IgG or IgM) that collectively were presented as heat maps of signal intensity.

### Histopathology

For histological analysis, intestines were cut longitudinally, rolled by the “Swiss roll” method (38), fixed in formalin, embedded in paraffin, sectioned through the central part of the roll and stained with hematoxylin and eosin. The stained slides were scanned with a Leica SCN400 F whole-slide scanner, and the images were analyzed using the SlidePath Digital Image Hub. Histological sections were then scored on a 0–4 scale with 0.5 increments according to the following criteria: 0, intact crypt architecture, normal epithelium with goblet cells, no neutrophil infiltration; 1, patchy neutrophil infiltration, occasional epithelial dysplasia; 2, foci of neutrophils, occasional cryptitis, epithelial damage; 3, diffuse leukocyte infiltration, crypt abscesses (debris and neutrophils inside of lumen), transmural infiltration to visceral peritoneum, crypt dropout; 4, severe loss of tissue architecture, widespread crypt damage.

### Gene expression analysis

For gene expression analysis of colon tissue, fragments of large intestine measuring 1 × 1 mm (30–35 mg) were homogenized in TRIzol (Thermo Fisher) using Lysing Matrix D beads (MP Biomedical) and a Fast-Prep-24 Instrument tissue homogenizer (MP Biomedical). Spleen single cell suspensions were also lysed in the presence of TRIzol, and the RNA was isolated after precipitation with chloroform and washing with 100% ethanol. After reverse transcription with SuperScript III (ThermoFisher), quantitative PCR of cDNA was performed using SYBR green (Roche) on a Bio-Rad CFX96 Touch™ instrument.

### Statistics

Data are represented as median with the distribution of all individual animals depicted. The data are pooled from at least 3 independent experiments. Unless noted otherwise, sample distribution was analyzed by the nonparametric Kruskal-Wallis test, and in cases when significance was reached with Kruskal-Wallis test, each group was individually compared using the *post-hoc* Dunn's multiple comparison test. The significance is defined as follows ^*^P ≤ 0.05, ^**^P ≤ 0.01, ^***^P ≤ 0.001.

## Results

### *Tcf4* haplodeficiency does not prevent aberrant immune activation caused by *Was* deficiency

**Figure 1:**
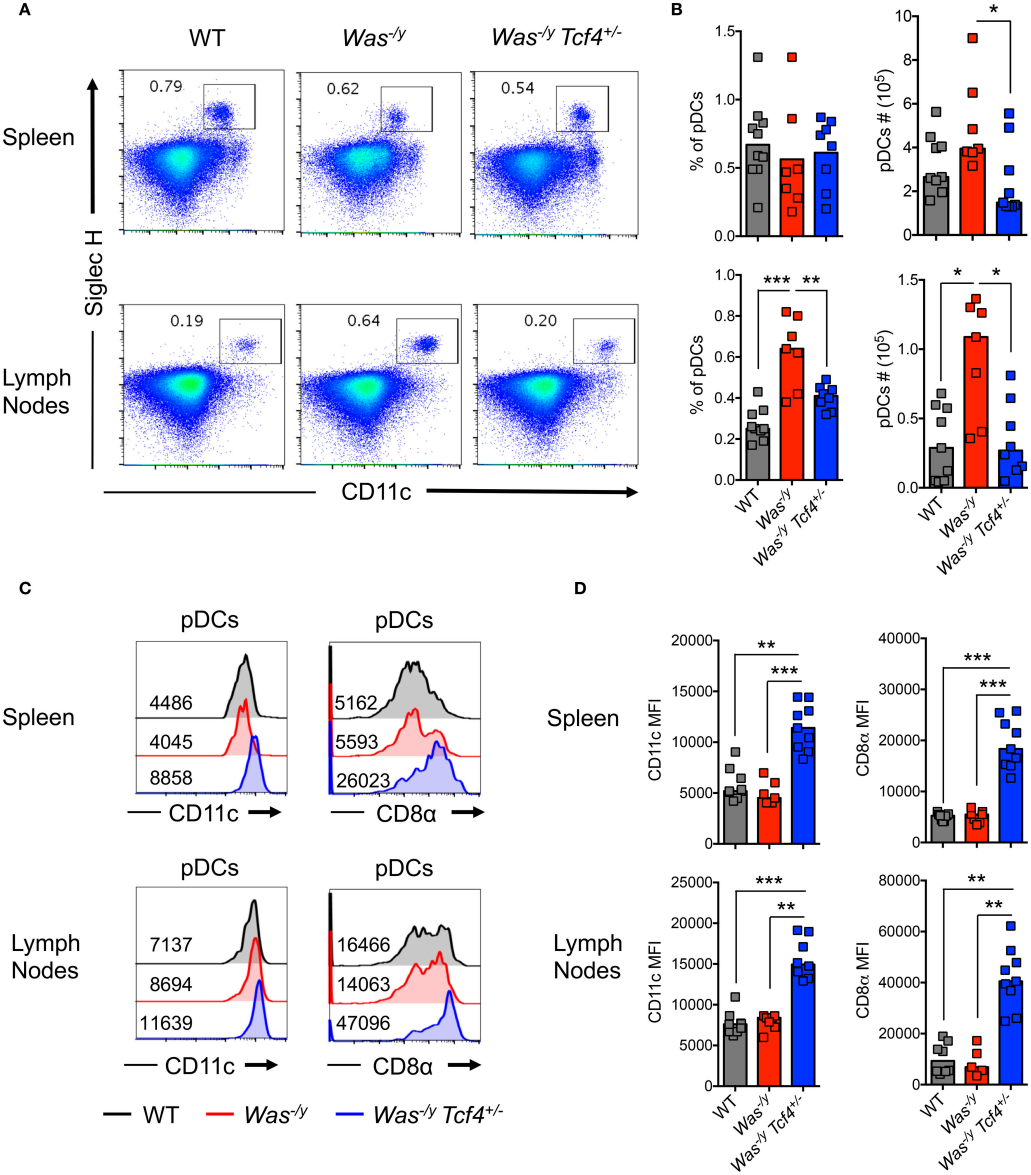
Reduced numbers and altered phenotype of pDCs upon *Tcf4* haplodeficiency in *Was*-deficient mice.WT, *Was*^−/y^ or *Was*^−/y^ *Tcf4*^+/−^ mice were analyzed at 30 weeks of age. **(A)** pDCs were identified by flow cytometry analysis of CD11c and Siglec H expression from CD11b^−^ live cells in the spleen and LNs. The frequency of pDCs is indicated in each plot. **(B)** Frequency and absolute numbers of pDCs (CD11b^−^ CD11c^+^ Siglec H^+^ pDCs among total live cells) in the spleen and LN of individual mice are shown. Horizontal bars indicate median value. Data were pooled from 4 independent experiments. **(C)** CD11c and CD8α expression with the mean fluorescent intensity (MFI) indicated on pDCs (CD11b^−^ CD11c^+^ Siglec H^+^) from the spleen and LN as analyzed by flow cytometry. **(D)** MFI of CD11c and CD8α on pDCs from the spleen and LN of each individual mice as pooled from 4 independent experiments. Horizontal bars indicate the median. Statistical significance estimated by Kruskal-Wallis test followed by a *post-hoc* Dunn's multiple comparison test is indicated as follows ^*^P ≤ 0.05, ^**^P ≤ 0.01, ^***^P ≤ 0.001.

In order to evaluate pDC function in autoimmunity mediated by *Was* deficiency, we crossed *Tcf4*^+/−^ haplodeficient males (129Sv background) with *Was*^−/−^ females (C57BL/6J background). As *Was* is located on the X chromosome, the F_1_ generation males obtained on the hybrid (B6/129Sv)F_1_ background are deficient for WASP (*Was*^−/y^) and either proficient or haplodeficient for *Tcf4*. We observed a similar frequency of pDCs, defined by the expression of CD11c and Siglec H (Figure 1A), in the spleen of *Was*^−/y^ *Tcf4*^+/−^ animals compared to *Was*^−/y^ and wild type (WT) animals on the same hybrid background (Figure 1B) However, the absolute number of pDCs within the spleen of *Was*^−/y^ *Tcf4*^+/−^ mice was significantly reduced compared to *Was*^−/y^ mice (Figure 1B). Within lymph nodes (LN), pooling axillary, inguinal and cervical LN, both the frequency and number of pDCs were significantly increased in *Was*^−/y^ mice compared to WT control but remained unchanged in *Was*^−/y^ *Tcf4*^+/−^ mice (Figure 1B). In the mesenteric LN (MLN) the frequency and numbers of pDCs in *Was*^−/y^ and *Was*^−/y^ *Tcf4*^+/−^ mice were increased compared to control animals with a trend toward a lower number of pDCs in *Was*^−/y^ *Tcf4*^+/−^ mice (Figures S1A,B). Haplodeficiency of *Tcf4* was previously shown to alter the phenotype of pDCs, which was associated with an inability to produce IFN-I (32, 39). In agreement with these reports we observed that pDCs from *Was*^−/y^ *Tcf4*^+/−^ mice in the spleen and LN express higher levels of cDC specific markers such as CD11c and CD8α (Figure 1C). The increased expression of CD11c and CD8α was consistently observed among all *Was*^−/y^ *Tcf4*^+/−^ mice and was significantly higher than in *Was*^−/y^ and WT animals (Figure 1D). Similarly, pDCs from the MLNs of *Was*^−/y^ *Tcf4*^+/−^ mice upregulated CD11c and CD8α on their surface (Figures S1C,D). The impact of *Tcf4* haplodeficiency was specific to pDCs and did not affect the overall distribution of cDCs in the spleen, LN, and MLN of *Was*-deficient animals (Figure S1E). Thus, our results indicate that *Tcf4* haplodeficiency reduces pDC numbers in *Was*-deficient mice and strongly impairs their phenotype, further validating *Tcf4* haplodeficiency as a pDC-specific tool.

**Figure 2:**
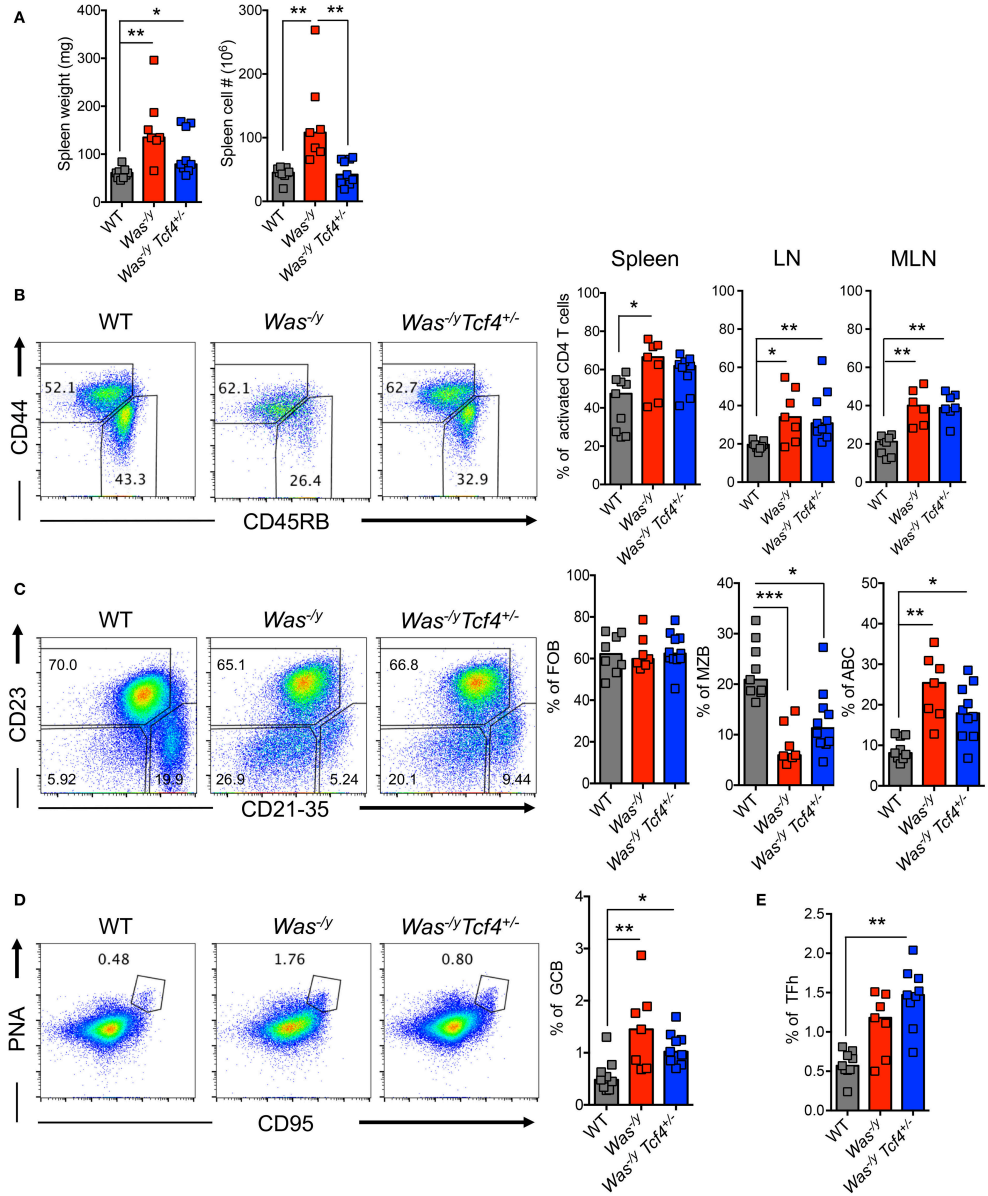
No change in overall immune activation upon *Tcf4* haplodeficiency in *Was*-deficient mice. Analysis of WT, *Was*^−/y^ or *Was*^−/y^ *Tcf4*^+/−^ mice at 30 weeks of age. **(A)** Spleen weight and cellularity were measured from individual mice. **(B–E)** T and B lymphocyte populations within the spleen were analyzed by flow cytometry. **(B)** The frequency of both naïve (CD44^−^, CD45RB^+^) and activated (CD44^+^, CD45RB^−^) cells among live CD4^+^ T cells was determined in the spleen, LN and MLN of the indicated animals. **(C)** The frequency of follicular B cells (FOB, CD23^+^, CD21^−^), marginal zone B cells (MZB, CD23^−^, CD21^+^) and age associated B cells (ABC, CD23^−^, CD21^−^) among mature B220^+^ CD93^−^ B cells was measured. **(D)** Analysis of germinal center B cells (GCB, PNA^+^ and CD95^+^ among total B220^+^ B cells) and **(E)** T follicular helper cell (TFh, CXCR5^+^, PD1^+^, and BCL6^+^ among CD4^+^ T cells) frequencies. Values from individual animals are shown and horizontal bars represent median. Data were pooled from 4 independent experiments and statistical significance estimated by Kruskal-Wallis test followed by a *post-hoc* Dunn's multiple comparison test is indicated as follows ^*^P ≤ 0.05, ^**^P ≤ 0.01, ^***^P ≤ 0.001.

We explored the impact of pDC impairment due to *Tcf4* haplodeficiency on the phenotype of *Was*-deficient animals. The splenomegaly in *Was*^−/y^ mice showed a trend toward reduction in *Was*^−/y^ *Tcf4*^+/−^ mice, which reached significance for total splenocyte numbers (Figure 2A). We then analyzed the activation status of CD4^+^ T cells by the expression of CD45RB (naïve T cells) and CD44 (activated T cells) markers. We observed that *Was*^−/y^ and *Was*^−/y^ *Tcf4*^+/−^ mice exhibited higher frequency of activated CD4^+^ T cells in comparison to control WT animals (Figure 2B). Similar results were observed in LNs and MLNs, indicating a systemic activation of CD4^+^ T cells in *Was*^−/y^ animals regardless of *Tcf4* status (Figure 2B). However, no defect in CD8^+^ T cell number and activation profile was detected (not shown). In addition to CD4^+^ T cell activation, an increased frequency and number of regulatory CD4^+^ T cells expressing the transcription factor forkhead box P3 (Foxp3) was observed in *Was*^−/y^ and *Was*^−/y^ *Tcf4*^+/−^ mice, most likely in response to the aberrant immune activation in these mice (Figure S2A). *Was*-deficient mice were previously described to display an abnormal distribution of peripheral B cells including loss of a marginal zone B (MZB) cell population (40). Consistent with this report, *Was*^−/y^ mice showed strongly reduced frequency and numbers of CD21/35^+^ CD23^−^ of MZB cells with no change in the population of CD23^+^ CD21/35^−^ follicular B cells (FOB) (Figure 2C, Figure S2B). The loss of MZB cells was not rescued by specific pDC ablation in *Was*^−/y^ *Tcf4*^+/−^ mice (Figure 2C, S2B). Another feature of multiple autoimmune strains is the accumulation of an unusual population of age-associated B cells (ABCs), negative for both FOB (CD23) and MZB (CD21/35) markers (41, 42). The frequency of ABCs in both *Was*^−/y^ and *Was*^−/y^ *Tcf4*^+/−^ mice was significantly increased compared to WT controls (Figure 2C). However, the absolute numbers in *Was*^−/y^ *Tcf4*^+/−^ remained comparable to WT mice (Figure S2B). In addition to the altered frequency of different B cell subsets, we observed that FOB and ABC cells from *Was*^−/y^ and *Was*^−/y^ *Tcf4*^+/−^ mice displayed an activated phenotype as reflected by increased expression of CD40 (Figure S2C). Finally, *Was*^−/y^ mice had an increased frequency of germinal center B cells (GCB) characterized by the expression of CD95 (Fas) and peanut agglutinin (PNA) (Figure 2D). The increase in GC frequency was associated with an increase in CD4^+^ Tfh expressing the chemokine receptor CXCR5 and the transcription factor BCL6 (Figure 2E, Figure S2E). Although *Tcf4* haplodeficiency was associated with a reduction in the absolute number of GCB in the spleen compared to *Was*^−/y^ (Figure S2D) the frequency of GCB was still increased compared to WT controls (Figure 2D). As observed in *Was*^−/y^ animals, the frequency and absolute numbers of CD4^+^ Tfh cells were significantly increased in *Was*^−/y^ *Tcf4*^+/−^ animals compared to control (Figure 2E, Figure S2E). Overall, apart from decreased splenomegaly, Tcf4 haplodeficiency had no impact on the general immune activation caused by *Was*-deficiency.

### *Tcf4* haplodeficiency does not prevent autoantibody production in *Was*-deficient mice

**Figure 3:**
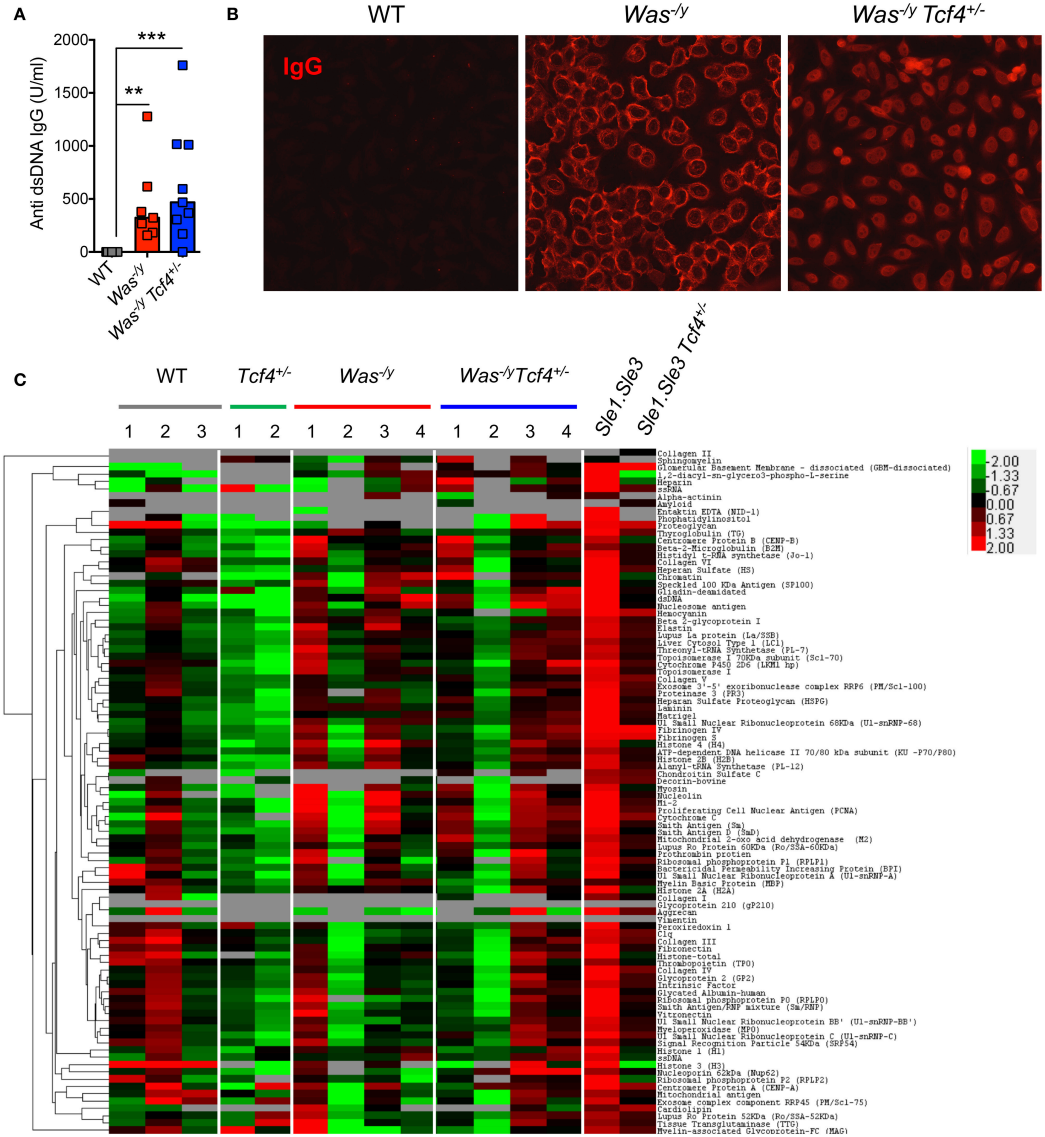
*Tcf4* haplodeficiency does not affect autoreactive antibody production in *Was*-deficient mice. Sera from WT, *Was*^−/y^, or *Was*^−/y^ *Tcf4*^+/−^ mice were obtained at 30 weeks of age and analyzed for autoreactive antibody production. **(A)** Anti-dsDNA IgG levels in the sera of indicated mice were determined by ELISA. Data were pooled from 4 independent experiments and bars represent the median. **(B)** ANA staining of IgG (red) using fixed Hep-2 cells following incubation with the sera of mice with the indicated genotype as analyzed by fluorescent microscopy. Images are representative of 2 independent staining experiments that include 6 animals for each genotype. **(C)** IgG autoreactivity in the indicated mice at 30 weeks of age as measured by an antigen array. Shown are heat maps of the relative IgG seroreactivity to the indicated antigens. Statistical significance estimated by Kruskal-Wallis test followed by a *post-hoc* Dunn's multiple comparison test is indicated as follows ^**^P ≤ 0.01, ^***^P ≤ 0.001.

Multiple features of autoimmunity have been observed in *Was*-deficient mice, including increased levels of autoreactive antibodies such as anti-dsDNA antibodies (40). We have previously observed that *Tcf4* haplodeficiency was able to reduce the autoreactive anti-RNA and anti-dsDNA antibodies secretion in two experimental models of SLE (13). Therefore, we sought to investigate if such *Tcf4* targeting may also impact the production of autoreactive antibodies in *Was*-deficient mice. We observed that compared to controls, *Was*^−/y^ animals showed significantly higher titers of auto-antibodies directed against double-stranded DNA (dsDNA) (Figure 3A). Specific targeting of pDCs in *Was*^−/y^ *Tcf4*^+/−^ mice did not affect the production of such autoantibodies (Figure 3A). The overall prevalence of antinuclear antibodies (ANA) was also increased in *Was*-deficient mice regardless of *Tcf4* status (Figure 3B). While the occurrence of ANA remained consistent between both groups, ANA staining from *Was*^−/y^ sera showed a perinuclear distribution while that of *Was*^−/y^ *Tcf4*^+/−^ sera was primarily nuclear (Figure 3B). This differential ANA profile prompted us to analyze the overall autoreactivity in those mice using an autoantigen microarray. While WT and *Tcf4*^+/−^ mice displayed very little autoreactive IgG with the exception of one outlier (mouse WT2), 3 out of 4 mice in the *Was*^−/y^ and *Was*^−/y^ *Tcf4*^+/−^ groups showed a broad range of autoreactive IgG specific to nucleic acids, multiple protein, and ribonucleoproteins (Figure 3C). In addition, these autoreactive IgG profiles detected across *Was*^−/y^ animals were largely unaffected by *Tcf4* haplodeficiency (Figure 3C). Similar results were observed from the quantification of autoreactive IgM (Figure S3). Importantly, *Tcf4* haplodeficiency in *Sle1.Sle3* lupus prone mice strongly reduced the levels of most autoreactive IgGs (Figure 3C), consistent with our previous observation (13). Collectively these results demonstrate that pDC impairment due to *Tcf4* haplodeficiency does not impact the production of autoantibodies in *Was*-deficient animals.

### *Tcf4* haplodeficiency does not affect colitis development in *Was*-deficient mice

**Figure 4:**
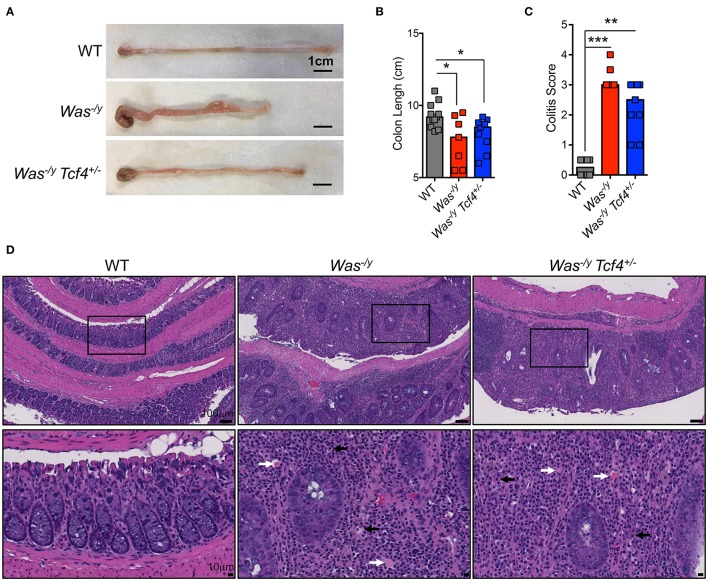
No significant impact of *Tcf4* haplodeficiency on colitis development in *Was*-deficient mice. WT, *Was*^−/y^ or *Was*^−/y^ *Tcf4*^+/−^ mice were analyzed at 30 weeks of age. **(A)** Representative pictures of colon (scale bars, 1 cm) and **(B)** colon lengths from individual mice of the indicated genotype are shown. Bars represent median value. Data were pooled from 4 independent experiments. **(C)** Histopathological scores of the colon pathology were determined from individual indicated animals. Bars represent the median, and data were pooled from 3 independent experiments. **(D)** Colon paraffin sections of the indicated mice were stained by hematoxylin and eosin (H&E) and analyzed by microscopy. Upper panels taken at 10X magnification (scale bar, 100 μm) and lower panels show a 40X magnification (scale bar, 10 μm) of the indicated area (square). In the magnified panels, black arrows indicate plasma cells and white arrows Russell bodies. Images are representative of at least 5 animals in each group from 3 independent experiments. Statistical significance estimated by Kruskal-Wallis test followed by a *post-hoc* Dunn's multiple comparison test is indicated as follows ^*^P ≤ 0.05, ^**^P ≤ 0.01, ^***^P ≤ 0.001.

In addition to systemic autoimmunity, WASP-deficient individuals (10%) and mice (100%) develop spontaneous colitis (43). Colitis development in animals lacking WASP is mediated by CD4^+^ T cells (44) and facilitated by innate immune cells (45). Therefore, we studied whether pDC impairment affects colitis development in *Was*-deficient mice. Mice were analyzed at 6 months of age, and at that time they did not show rectal prolapse, rectal bleeding or major weight loss (not shown). However, the colon length of *Was*^−/y^ mice was significantly shorter than that of WT control animals (Figures 4A,B). The reduction in colon length, which is a sign of colonic inflammation, was similar in *Was*^−/y^ *Tcf4*^+/−^ mice (Figures 4A,B). Histological analysis of the large intestine of *Was*^−/y^ and *Was*^−/y^ *Tcf4*^+/−^ mice showed severe colitis (Figure 4C). Irrespectively of *Tfc4*-haplodeficiency, colons of *Was*-deficient animals exhibited diffuse leukocyte infiltration, epithelial dysplasia and crypt dropout (Figure 4D). While CD4^+^ T cells were previously described as the main constituents of this leukocyte infiltration [43], we also noted an accumulation of plasma cells (black arrows) and Russell bodies (plasma cells accumulating Ig, white arrows) in the colon of *Was*^−/y^ and *Was*^−/y^ *Tcf4*^+/−^ mice (Figure 4D). Similar levels of colonic inflammation were observed in mice that were analyzed at 10 months of age (not shown). These results indicate that *Tcf4* haplodeficiency, which specifically impairs pDC numbers and function, does not prevent the development of colitis in *Was*-deficient mice. The expression of IFN-inducible marker Sca-1 on lymphocytes is a faithful marker of aberrant IFN signaling (46) and was specifically reduced by *Tcf4* haplodeficiency in SLE-prone mice (13). In contrast, we did not observe any increase in Sca1 expression on T and B cells from *Was*^−/y^ mice compared to WT controls (Figure S4A). In addition, the expression profile of IFN-I inducible genes in the spleen (Figure S4B) and the colon (Figure S4C) were similar in WT control mice and *Was*^−/y^ mice regardless of their *Tcf4* status. Overall, genetic impairment of pDCs did not affect autoimmunity or IBD caused by *Was* deficiency suggesting that these cells are largely dispensable in this model.

### Conditional deletion of *Tcf4* depletes pDCs in *Il10*-deficient mice

**Figure 5:**
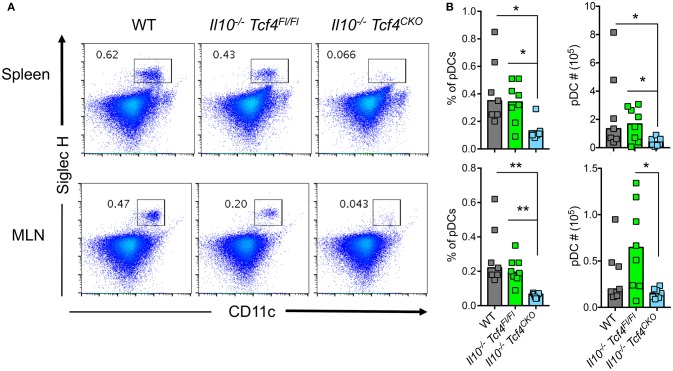
DC-specific deletion of *Tcf4* significantly depletes pDCs in *Il10*-deficient mice. *Il10*^−/−^ *Itgax*-Cre^−^ *Tcf4*^flox/flox^ (*Il10*^−/−^ *Tcf4*^Fl/Fl^) or *Il10*^−/−^ *Itgax*-Cre^+^ *Tcf4*^flox/flox^ (*Il10*^−/−^*Tcf4*^*CKO*^) littermates with DC-specific *Tcf4* CKO were analyzed along WT controls at 15 weeks of age. **(A,B)** pDCs were analyzed by flow cytometry of cells from the spleen and MLN. The frequency of CD11b^−^ CD11c^+^ Siglec H^+^ pDCs is indicated in each plot. **(B)** Frequency and absolute numbers of CD11b^−^ CD11c^+^ Siglec H^+^ pDCs among total live cells in the spleen and MLN of individual animals are shown, and horizontal bars indicate the median. Data represent 3 independent experiments. Statistical significance estimated by Kruskal-Wallis test followed by a *post-hoc* Dunn's multiple comparison test is indicated as follows ^*^P ≤ 0.05, ^**^P ≤ 0.01.

Because *Tcf4* haplodeficiency results in only a partial impairment of pDCs, we sought to examine the effect of a more profound pDC depletion on colitis. To this end, we used mice in which conditional (floxed) alleles of *Tcf4* are combined with a DC-specific *Itgax* (CD11c)-Cre deleter strain (35). As *Tcf4* is not expressed by cDCs, *Itgax*-Cre mediated deletion of *Tcf4* affects only pDCs and causes their profound constitutive depletion (15). We crossed the DC-specific *Tcf4* conditional knockout (CKO) strain with *Il10*-deficient mice (*Il10*^−/−^*)* that develop colitis resembling human IBD (33). Although colitis in *IL10*-deficient humans develops early in childhood, in *Il10*-deficient mice on pure C57BL/6J background, colitis only occurs in adult animals and in a milder form of disease than observed in other genetic backgrounds [47]. We next analyzed *Il10*^−/−^ *Itgax*-Cre^−^ *Tcf4*^flox/flox^ (*Il10*^−/−^ *Tcf4*^Fl/Fl^), *Il10*^−/−^ *Itgax*-Cre^+^ *Tcf4*^flox/flox^ (*Il10*^−/−^ *Tcf4*^CKO^), and *Il10*^+/+^ *Tcf4*^Fl/Fl^ (WT) control mice at 3 months of age to evaluate the impact of such targeting on pDC distribution. The frequency of pDCs was consistently reduced in the spleen and MLNs of *Il10*^−/−^ *Tcf4*^CKO^ mice compared to both WT and *Il10*^−/−^ *Tcf4*^Fl/Fl^ controls (Figures 5A,B). This reduced frequency was associated with significantly lower absolute numbers of pDCs in the spleen and MLNs of *Il10*^−/−^ *Tcf4*^CKO^ mice compared to controls (Figure 5B). Although conditional loss of *Tcf4* had a slight effect on the frequency of overall cDCs in the spleen compared to WT controls, there was no impact on the absolute numbers or other detectable change in the subsets of cDCs (Figures S5A,B). An increased number of cDCs as well as variation in the frequency and numbers of cDC subsets were observed in the MLN upon *Il10* deficiency and were independent of the conditional loss of *Tcf4* (Figure S5B). Thus, conditional deletion of *Tcf4* in mice that are deficient for *Il10* causes specific and constitutive pDC ablation.

### pDC depletion does not affect the aberrant immune activation in *Il10-*deficient mice

**Figure 6:**
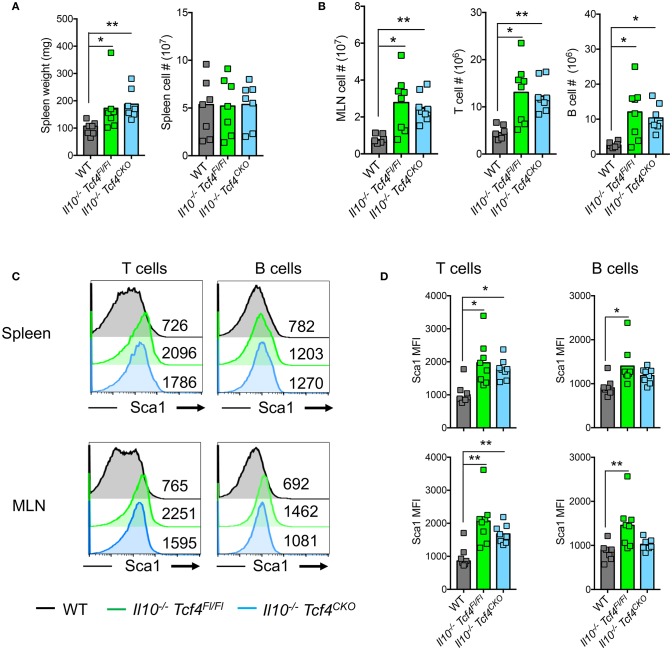
DC-specific deletion of *Tcf4* does not impact the overall immune activation in *Il10*-deficient mice. *Il10*^−/−^ *Itgax*-Cre^−^ *Tcf4*^flox/flox^ (*Il10*^−/−^ *Tcf4*^Fl/Fl^) or *Il10*^−/−^ *Itgax*-Cre^+^ *Tcf4*^flox/flox^ (*Il10*^−/−^*Tcf4*^*CKO*^) littermates with DC-specific *Tcf4* CKO were analyzed along WT controls at 15 weeks of age. **(A)** Spleen weight and cellularity of individual animals were measured. **(B)** MLN cellularity and the absolute numbers of (TCRß^+^) T cells and (B220^+^) B cells were determined by flow cytometry from individual mice. **(C,D)** Sca1 expression on (TCRß^+^) T cells and (B220^+^) B cells from the spleen and MLNs was assessed by flow cytometry. **(C)** Histograms show representative analysis of Sca1 expression and the MFI of Sca1 is indicated in each plot and **(D)** plots show Sca1 MFI of individual animals. In all plots, horizontal bars represent the median. Data for all panels pooled from 3 independent experiments. Statistical significance estimated by Kruskal-Wallis test followed by a *post-hoc* Dunn's multiple comparison test is indicated as follows ^*^P ≤ 0.05, ^**^P ≤ 0.01.

In contrast to *Was*-deficient mice, *Il10*^−/−^ mice do not show major signs of systemic autoimmunity. Indeed, T and B cells develop normally and no aberrant production of autoantibodies is detected in mice that lack IL-10 [33]. Although *Il10*^−/−^ *Tcf4*^Fl/Fl^ and *Il10*^−/−^ *Tcf4*^CKO^ animals had a minor splenomegaly compared to WT controls, there was no difference in spleen cellularity (Figure 6A). However, *Il10*^−/−^ *Tcf4*^Fl/Fl^ mice consistently showed an increase in MLN cell numbers that is associated with intestinal inflammation and was also detected in *Il10*^−/−^ *Tcf4*^CKO^ animals (Figure 6B). The increased MLN cellularity was primarily associated with significantly increased numbers of T and B cells (Figure 6B). In addition, we observed a modest increase in CD4^+^ T cell activation in the MLN, but not the spleen, of *Il10*^−/−^ *Tcf4*^CKO^ mice (Figure S6A). Although alterations in the frequency of B cell subsets in the spleen were detected in *Il10*-deficient animals regardless of *Tcf4* status compared to WT control, the absolute numbers for each B cell subset show no major differences across all the analyzed mice (Figure S6B). Despite the trend of reduced frequency, the number of GCBs was significantly increased in the MLN in *Il10*^−/−^ *Tcf4*^CKO^ animals due to the increased MLN cellularity (Figure S6B). T and B cells both in the spleen and MLN of *Il10*^−/−^ *Tcf4*^Fl/Fl^ mice showed increased expression of Sca1, and such increased Sca1 expression was also found in *Il10*^−/−^ *Tcf4*^*CKO*^ mice (Figures 6C,D). Sca1 was previously described to be a reliable marker of elevated IFN signaling (46), and we have recently confirmed that Sca1 expression is abolished in *Ifnar*-deficient T and B cells (our unpublished data). Therefore, the induction of Sca1 in *Il10*^−/−^ mice independently of *Tcf4* ablation suggests that pDCs are not the major source of the aberrant IFN-I signaling induced by *Il10* deficiency. These results demonstrate that profound pDC depletion does not affect the immune activation observed in *Il10*-deficient mice.

### pDC depletion does not prevent the development of colitis in *Il10*-deficient mice

**Figure 7:**
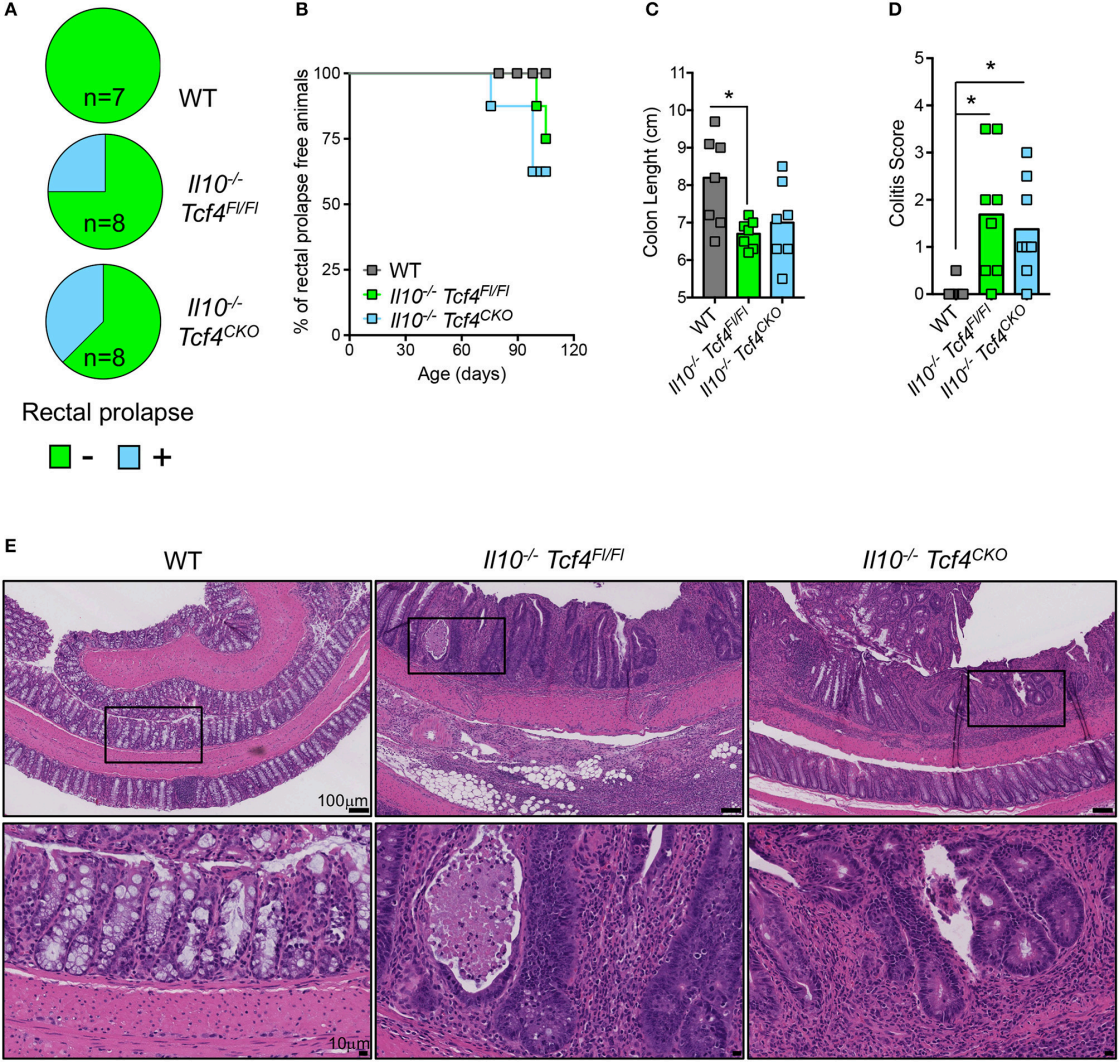
DC-specific deletion of *Tcf4* does not prevent colitis development in *Il10*-deficient mice. *Il10*^−/−^ *Itgax*-Cre^−^ *Tcf4*^flox/flox^ (*Il10*^−/−^ *Tcf4*^Fl/Fl^) or *Il10*^−/−^ *Itgax*-Cre^+^ *Tcf4*^flox/flox^ (*Il10*^−/−^*Tcf4*^*CKO*^) littermates with DC-specific *Tcf4* CKO were analyzed along WT controls at 15 weeks of age. **(A)** Rectal prolapse in animals of the indicated genotype. Data are represented as pie charts indicating the frequency of animals with (blue) and without (green) rectal prolapse. **(B)** Kaplan-Meier plot indicating the percentage of rectal prolapse free mice over time in the indicated genotype (*n* = 8/genotype). **(C)** Colon lengths from individual mice of the indicated genotype. Data were pooled from 3 independent experiments and horizontal bars represent the median. **(D)** Histopathological scores of the colon pathology were determined from individual indicated animals. Bars represent the median and data were pooled from 3 independent experiments. **(E)** Colon paraffin sections of the indicated mice were stained by H&E and analyzed by microscopy (scale bars, 100 μm). Upper panels represent a 10X magnification (scale bar, 100 μm) and lower panels show a 40X magnification (scale bar, 10 μm) of the indicated area (square). Images are representative of 6 animals in each group from 3 independent experiments. Statistical significance estimated by Kruskal-Wallis test followed by a *post-hoc* Dunn's multiple comparison test is indicated as follows ^*^P ≤ 0.05.

As expected, *Il10* deficiency was associated with the development of rectal prolapse. Indeed, 2 out of 8 *Il10*^−/−^ *Tcf4*^Fl/Fl^ mice and 3 out 8 *Il10*^−/−^ *Tcf4*^CKO^ mice showed rectal prolapse, while none of the 7 WT control mice did (Figure 7A). Rectal prolapse only occurred in adult animals when they were older than 2.5 months (Figure 7B). In addition, *Il10*^−/−^ *Tcf4*^Fl/Fl^ mice displayed a significant shortening of their colon, indicating the presence of colonic inflammation (Figure 7C). Although more variability among animals was observed, a trend for reduced colon length was also observed in *Il10*^−/−^ *Tcf4*^CKO^ mice (Figure 7C). We next performed histological analysis of the colon sections and observed a significantly increased pathological scores in both *Il10*^−/−^ *Tcf4*^Fl/Fl^ and *Il10*^−/−^ *Tcf4*^CKO^ animals compared to WT ones (Figure 7D). Colon pathology in *Il10*^−/−^ *Tcf4*^Fl/Fl^ and *Il10*^−/−^ *Tcf4*^CKO^ mice included massive leukocyte infiltration, neutrophils within the epithelium and lumen, crypt abscesses, epithelial dysplasia, and crypt damage (Figure 7E). Collectively, these histological features are characteristic of the substantial colonic inflammation caused by loss of IL-10, and their development was unaffected by specific pDC depletion.

## Discussion

We have explored the function of pDCs in IBD pathogenesis in mice that lack WASP or IL-10 and consequently spontaneously develop colitis. We observed that specific abrogation of pDCs does not impact the development of IBD *in vivo* in either model. The specific deletion of pDCs was achieved through targeting of the transcription factor TCF4, which is the master regulator of pDC fate and maintenance (32, 39). Indeed, we established a global monoallelic deletion of *Tcf4* in *Was*-deficient mice that causes a constitutive reduction in pDC numbers and a DC-specific loss of *Tcf4* in *Il10-deficient* mice inducing a complete and constitutive pDC abrogation. Such targeting of pDCs has been extensively analyzed previously. Indeed, we have shown that *Tfc4* haplodeficiency strongly impairs pDC ability to produce IFN-I in response to CpG-ODN A (32) and pushes them toward a “cDC-like” phenotype (32, 39). In addition, conditional ablation of *Tcf4* was also reported to reduce pDCs numbers and ablate pDCs-driven IFN-I response upon viral infections (48). The great majority of these studies revealed that cDCs are not affected by *Tcf4* haplodeficiency nor its complete conditional ablation, demonstrating the specificity of this system for targeting pDCs. *Tcf4* haplodeficiency and conditional targeting were also shown to ameliorate the development of SLE (13) and type 1 diabetes (15) *in vivo*, confirming key roles for pDCs in such pathologies. Finally, an identical *Tcf4* targeting was also used to rule out the involvement of pDCs in an experimental model of psoriasis (49), and we now extend these observations to genetic models of IBD.

WASP regulates leukocyte actin dynamics, controlling migration and a variety of effector functions (50). Deficiency in WASP causes the X-linked Wiskott-Aldrich syndrome that is associated with aberrant systemic immune activation and colitis in 10% of individuals. The aberrant systemic autoimmunity is recapitulated in mice that lack WASP, which unlike humans develop colitis with 100% penetrance, thus representing a relevant experimental model of IDB. While *Tcf4* haplodeficiency in *Was*-deficient mice specifically reduced pDC numbers it did not affect the heightened activation of immune responses or the development of colitis. These results differ from observations obtained in animal models of SLE in which pDC targeting ameliorated the overall pathology (12, 13). The observed difference in the effect of pDC ablation between these two diseases models is likely due to T and B cell-intrinsic functions of WASP. Indeed, conditional *Was*-deficiency in B cells is sufficient for the induction of B cell hyperactivation, loss of MZB and aberrant production of autoantibodies directed against a variety of self-molecules including DNA (40). Although, TCF4 is also expressed at low levels in B cells we did not observe significant differences in B cell phenotype and function between *Was*^−/y^ *and Was*^−/y^ *Tcf4*^+/−^ mice ruling out B cell-intrinsic effect of *Tcf4* haplodeficiency. This conclusion is also supported by our previous observation and two recently published reports showing respectively that *Tcf4* haplodeficiency (32) and conditional deletion specifically in B cells (51, 52) did not affect their overall development, phenotype or function. The absence of a B cell intrinsic TCF4 function is thought to be due to its redundancy with the homologous E protein E2a (encoded by *Tcf3*) (51, 52). In addition, WASP also regulates the function of T cells (43), particularly the suppressive potential of Tregs (53). Accordingly, transfer of *Was*-deficient CD4^+^ T cells into *Rag2*-deficient mice is sufficient to induce colitis (44). *Was*-deficient innate immune cells were also suggested to exacerbate colitis after the observation that the transfer of WT naïve CD4 T cells in *Was/Rag1* double-deficient mice induced a more severe colitis compared to their transfer into *Rag1*-deficient mice (45). It was recently shown that conditional *Was*-deficiency in macrophages but not in DCs (including pDCs) in the *Rag1*-deficient recipients led to the aggravation of such intestinal inflammation upon transfer of WT naïve CD4^+^ T cells (54). These results thus indicate that the IBD pathogenesis upon naïve CD4^+^ T cell transfer into *Was/Rag1* double-deficient mice is mainly driven by macrophages. However, the specific contributions of both macrophages and DCs to spontaneous colitis development in *Was*-deficient mice remains to be established. Finally, WAS patients were reported to display elevated serum levels of IFN-I as well as an IFN-I signature, reflected by the increased expression of IFN-I stimulated genes in PB leukocytes (29). IFN-I causing this signature was proposed to originate from pDCs after identifying that pDCs from WAS patients or *Was*-deficient mice were more responsive to TLR9 stimulation and as a result produced elevated levels of IFN-I (29). We were unable to detect an IFN-I signature as measured by the expression of IFN-I stimulated genes in *Was*-deficient animals and thus could not establish a link between pDCs and the aberrant IFN-I signature observed in WAS patients. These observations are consistent with previous reports showing that contrary to human SLE patients, murine models of SLE do not manifest a prominent IFN-I signature (13, 55) despite considerable evidence for IFN-I signaling in the pathogenesis of SLE *in vivo* (56, 57). Therefore, the lack of an observable IFN-I signature does not rule out the role of IFN-I and pDCs in murine models of autoimmunity. It is possible that a detectable IFN-I signature may have preceded the onset of disease (58) but was not present at late stages of the diseases (6 months) when we measured it in *Was*-deficient mice.

We also studied the role of pDCs during IBD pathogenesis in *Il10*-deficient mice. These mice are unable to produce the anti-inflammatory cytokine IL-10, which is essential for the control of immune responses and thus prevents colitis development. We specifically depleted pDCs using conditional targeting of both alleles of *Tcf4* mediated by *Itgax-*Cre in *Il10*^−/−^ mice. Although this strategy profoundly diminished the pool of pDCs in *Il10*^−/−^ mice, we did not detect any major impact on the development of colitis. The *Il10*^−/−^ mice are considered the gold standard colitis model that closely recapitulates human IBD. Mutations in *IL10* or the genes encoding its receptors *IL10RA/IL10RB* in humans were shown to cause autosomal recessive disease with CD-like colitis (59) and GWAS studies have identified associations between these three genes and sporadic IBD (60). While *IL10* and *IL10R* deficiency in humans induces a severe IBD that occurs early in childhood, the severity of IBD in *Il10*-deficent mice is strongly dependent on the genetic background as well as the husbandry conditions (47). In our study *Il10*-deficient mice were kept on a pure C57BL/6J background and housed in Specific Pathogen Free (SPF) conditions, both of which are factors associated with an attenuated IBD phenotype that is initiated only in adult animals. Therefore, our results provide strong genetic evidence in an experimental model of adult IBD with clear relevance to the human pathology that pDCs are dispensable in colitis pathogenesis. These results are in accordance with the observation that specific loss of *Il10ra* in macrophages is sufficient to phenocopy the global *Il10* deficiency (61). Indeed, IL-10 appears to act primarily on macrophages to prevent the development of colitis. This protective role of IL-10 was recently attributed to its ability to induce the metabolic reprogramming of macrophages subsequently preventing their aberrant production of IL-1β (62).

In contrast to our observations, pDCs were previously proposed to play an important role in colitis, exacerbating the overall IBD pathogenesis (29, 30). Using Siglec-H DTR knock in mice to inducibly deplete pDCs following DT treatment, Arimura *et al*. concluded that pDC depletion ameliorates DSS-induced colitis (30). In these settings pDCs were proposed to play an important role in the recruitment of pro-inflammatory monocytes and macrophages into the inflamed intestinal epithelium, consequently exacerbating the disease (30). Such discrepancies with our observations may be due to the use of different models of colitis and systems for pDC ablation. We used genetic IBD models that are T cell-dependent while Arimura et al. used T cell-independent DSS-induced colitis that arises from disruption of the intestinal barrier, which allows the entry of luminal microbes into the intestinal mucosa and consequently activates innate immune responses (63). Furthermore, Siglec H is not restricted to pDCs and was shown to be expressed on a subset of intestinal macrophages (31). Therefore, treatment of Siglec-H-DTR mice with DT should not only deplete pDCs but also a population of intestinal macrophages that may participate in the disease induced by DSS. In addition, pDCs were also suggested to contribute to development of IBD in *Was*-deficient mice (29). Prete et al. found that pDCs lacking *Was* produce heightened levels of IFN-I and that *Ifnar-*deletion in *Was*-deficient mice reduced the overall pathology including colitis (29). Although these results indicate an important role of IFN-I signaling in colitis development in *Was*-deficient mice, whether pDCs are the major source of this pathogenic IFN-I was not established. Our results demonstrating no impact of *Tcf4* specific targeting on the occurrence and the severity of colitis in *Was*-deficient animals argue against an important role of pDCs in this process.

Overall pDCs appear to be dispensable for disease development in common monogenic models of IBD. They may have a minor role in IBD that arises upon destruction of the intestinal barrier by environmental factors such as DSS. In this model pDCs do not appear to be the main effectors of IBD development, but rather exacerbate the disease [30]. Nevertheless, pDCs may contribute to the development of colitis when aberrantly activated. Accordingly, specific ablation of the inhibitory protein tyrosine phosphatase receptor type S (PTPRS) on pDCs was associated with the development of mild intestinal inflammation in mice (64). Lack of PTPRS on pDCs resulted in spontaneous IFN-I production by pDCs, which may be involved in establishing this inflammation. pDCs were also suggested to promote tolerance and ameliorate IBD upon stimulation by enteric viruses (65). While enteric viruses activate pDC production of IFN-I *ex-vivo* (65), direct *in vivo* evidence for pDC functions in the prevention of colitis development after infection with enteric viruses has not been demonstrated. Similarly, it was shown that IFN-I production resulting from CpG-ODN A stimulation protects mice from the development of experimental and spontaneous models of IBD induced by DSS treatment and *Il10*-deficiency, respectively (66, 67). The preventive impact of CpG-ODN A treatment on the overall IBD pathogenesis was shown to depend on IFNAR and TLR9 signaling (66, 67). However, whether pDCs play an important role in this process was not addressed. Therefore, it appears that IFN-I plays a controversial role in IBD, either contributing to the disease when aberrantly produced in a chronic manner or ameliorating the disease when acutely stimulated. The function of IFN-I in IBD thus needs further clarification as well as the role of pDCs as a source of such IFN-I. On the other hand, independently of IFN-I, pDCs were shown to mediate the protective role of PSA in IBD pathogenesis induced by intra-rectal administration of the haptenating agent 2,4,6-trinitrobenzene sulfonic acid (TNBS) (24). Indeed, selective depletion of pDCs following DT treatment of BDCA-2-DTR transgenic mice (68) abrogated the beneficial effect of PSA on IBD pathogenesis (24). This protective function of pDCs was only observed after PSA treatment whereas pDC depletion alone did not ameliorate colitis. These data indicate that pDCs are not essential in IBD pathogenesis, but their targeting by PSA may prevent colitis development, most likely through their ability to stimulate CD4^+^ T cell production of IL-10 (24).

Characterization of the deleterious role of pDCs in multiple autoimmune disorders such as SLE (12, 13), systemic sclerosis (14), and type I diabetes (15) has led to the development of novel therapeutic strategies aimed at specifically depleting pDCs (17). Our results indicate that such therapies may not benefit IBD patients. On the other hand, the observation that pDC-specific deletion does not impact IBD pathogenesis suggests that therapeutic pDC depletion will not be associated with off-target effects within the gastrointestinal tract of treated individuals.

## Author contributions

BR, MS, and RK initiated and supervised the project. CS, VS, MS, and BR designed experiments, analyzed, and interpreted results. CS and VS performed experiments with the help of LS, GJ, and AR. AN performed the histological analysis. The manuscript was written by CS, VS, and BR with the input from all authors.

### Conflict of interest statement

MS and RK were employees of Medimmune, LLC. The remaining authors declare that the research was conducted in the absence of any commercial or financial relationships that could be construed as a potential conflict of interest.
