# Supplementary material for: Plasmacytoid Dendritic Cells Are Largely Dispensable for the Pathogenesis of Experimental Inflammatory Bowel Disease
Source: Front Immunol. 2018 Oct 25;9:2475. doi: 10.3389/fimmu.2018.02475 (PMC6209677; doi:10.3389/fimmu.2018.02475)
Supplement: Supplementary file 1 [file Data_Sheet_1.pdf]

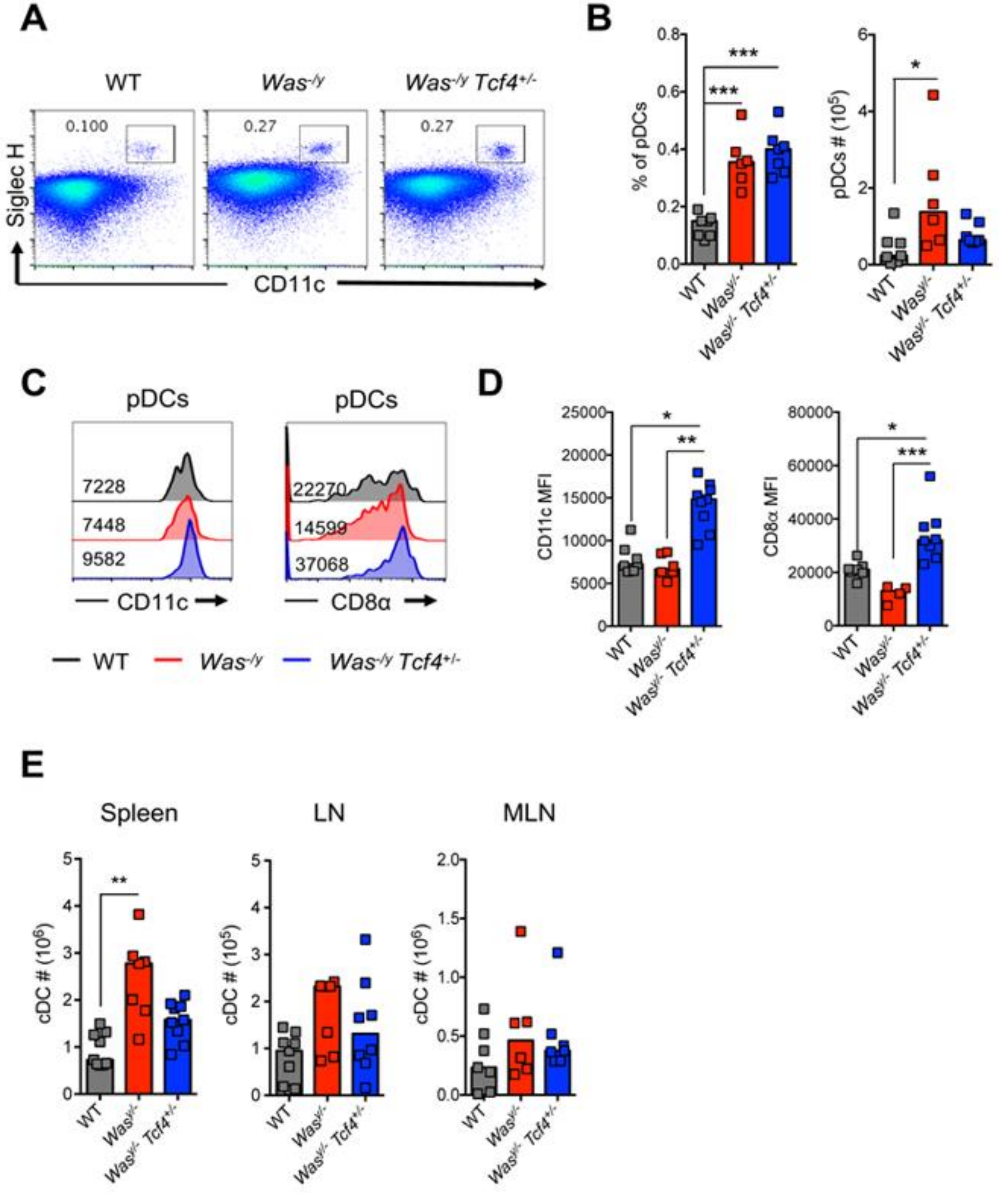


**FigS1.** Tcf4 haplodeficiency in Was-deficient mice alters the phenotype of pDCs in mesenteric lymph nodes and do not affect the overall distribution of cDCs

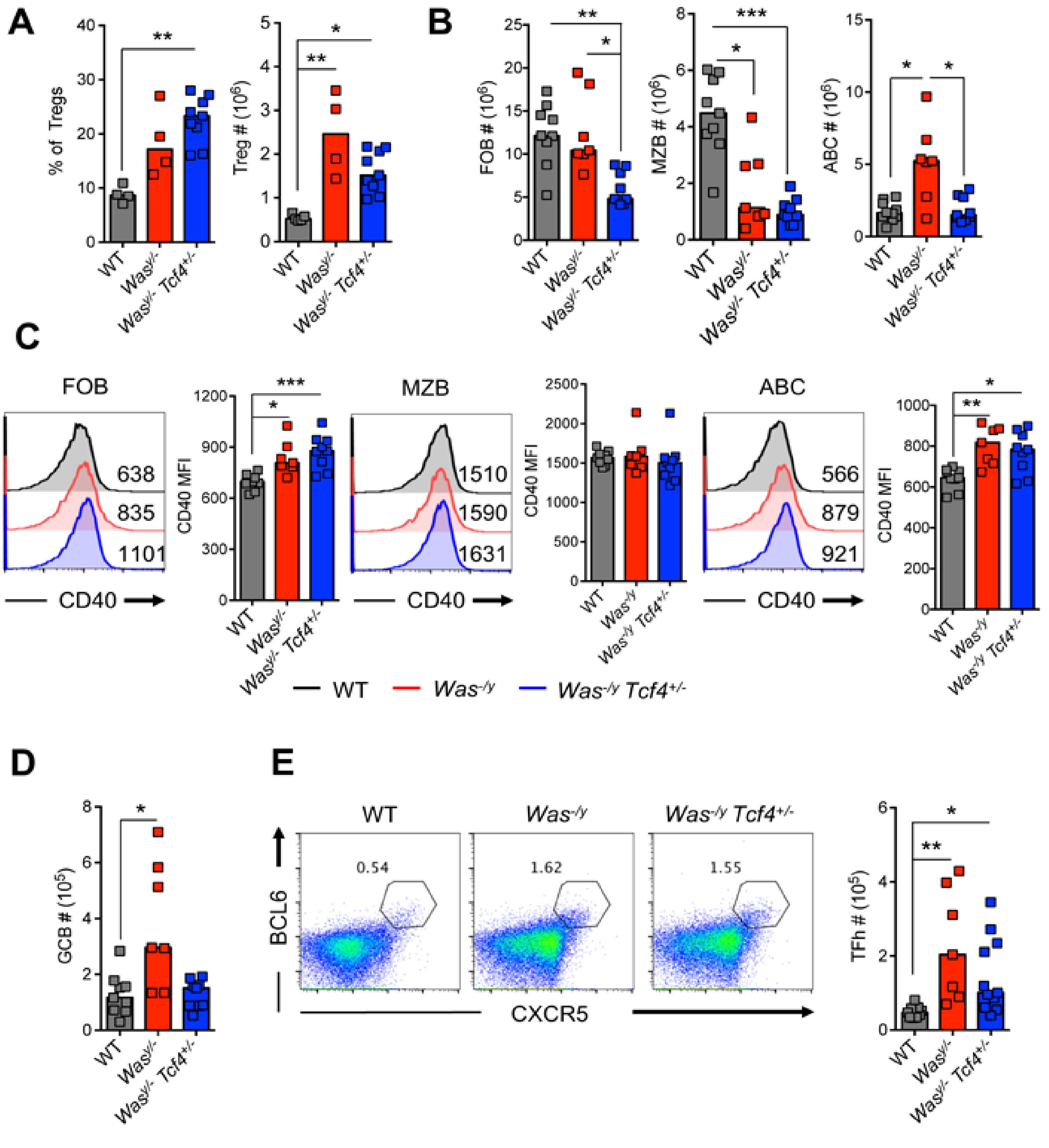


**FigS2.** *Tcf4* haplodeficiency does not impact the overall immune activation in *Was*-deficient mice

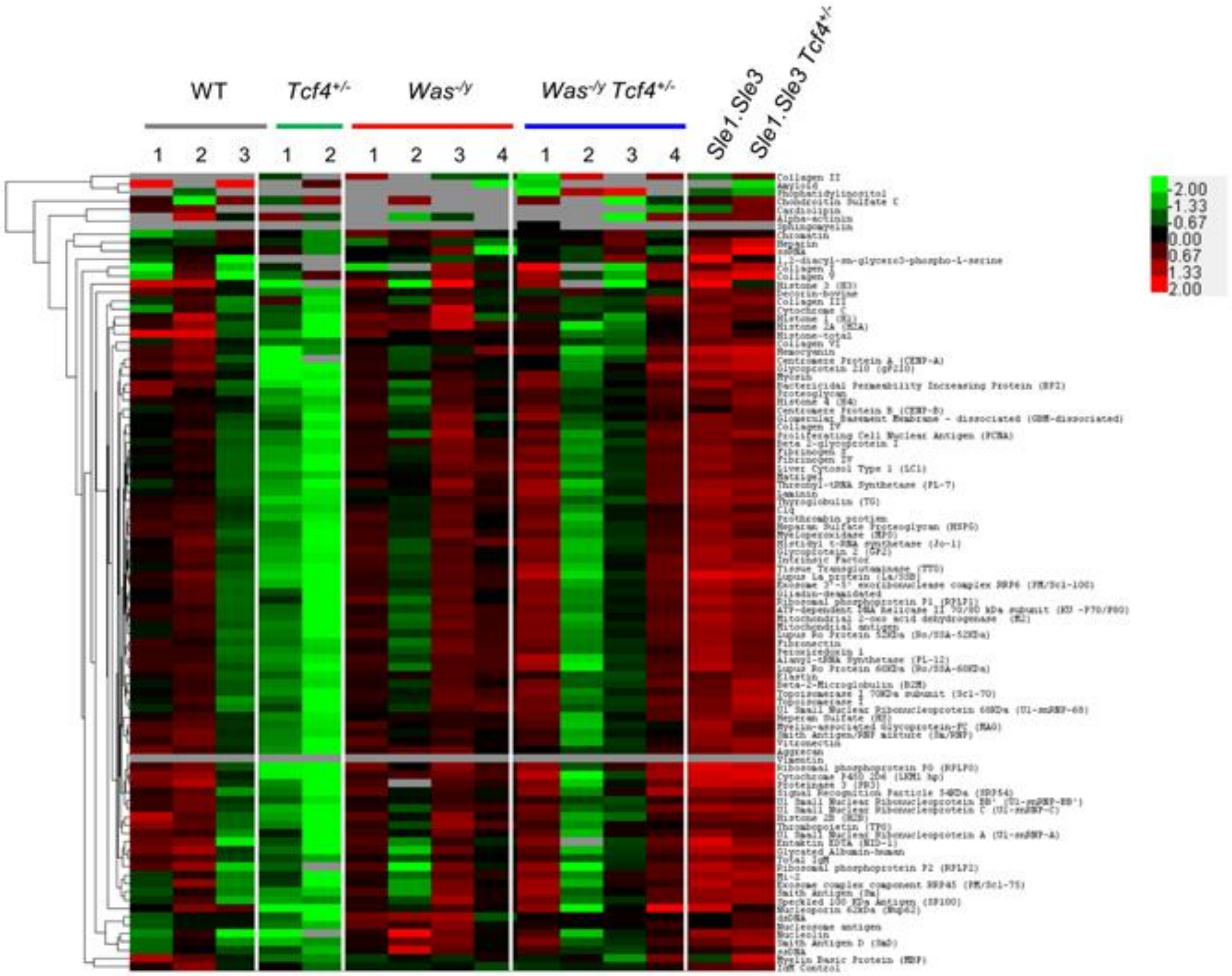


**FigS3.** *Tcf4* haplodeficiency does not affect autoreactive IgM production by *Was*-deficient mice

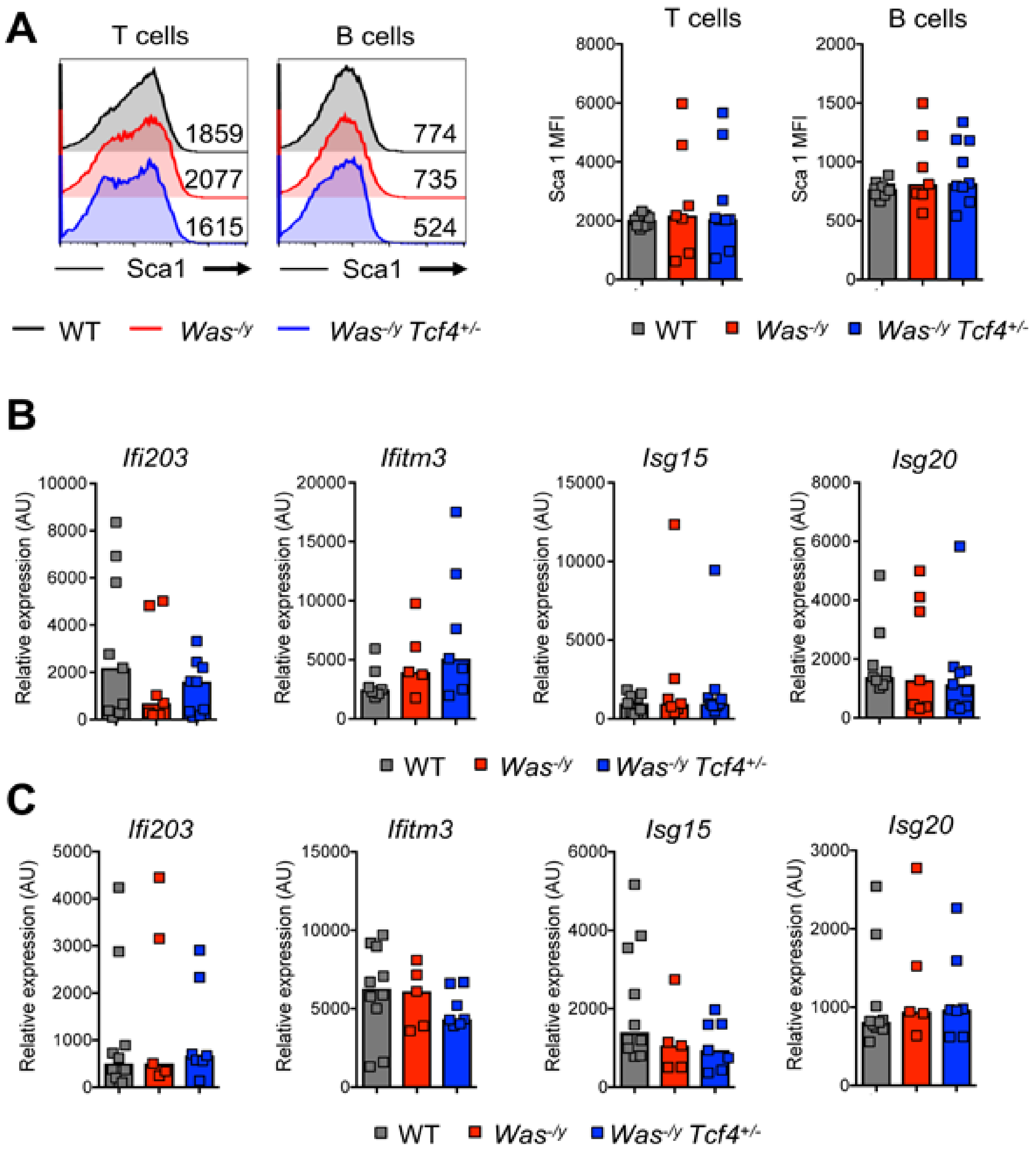


**FigS4.** *Was*-deficient mice do not show a type I interferon signature

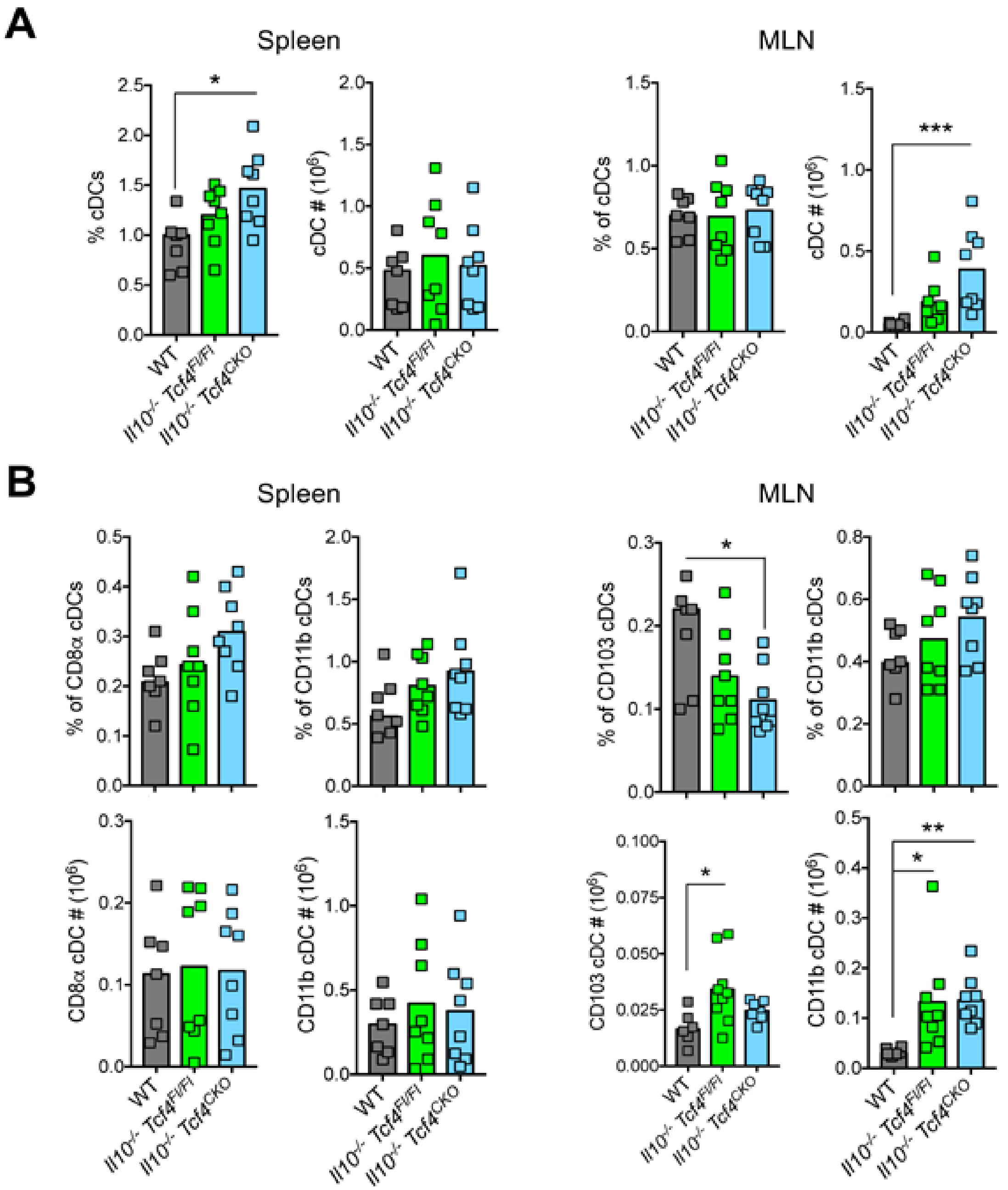


**FigS5.** DC-specific deletion of *Tcf4* does not impact the frequency and numbers of conventional DCs in *Il10*-deficient mice.

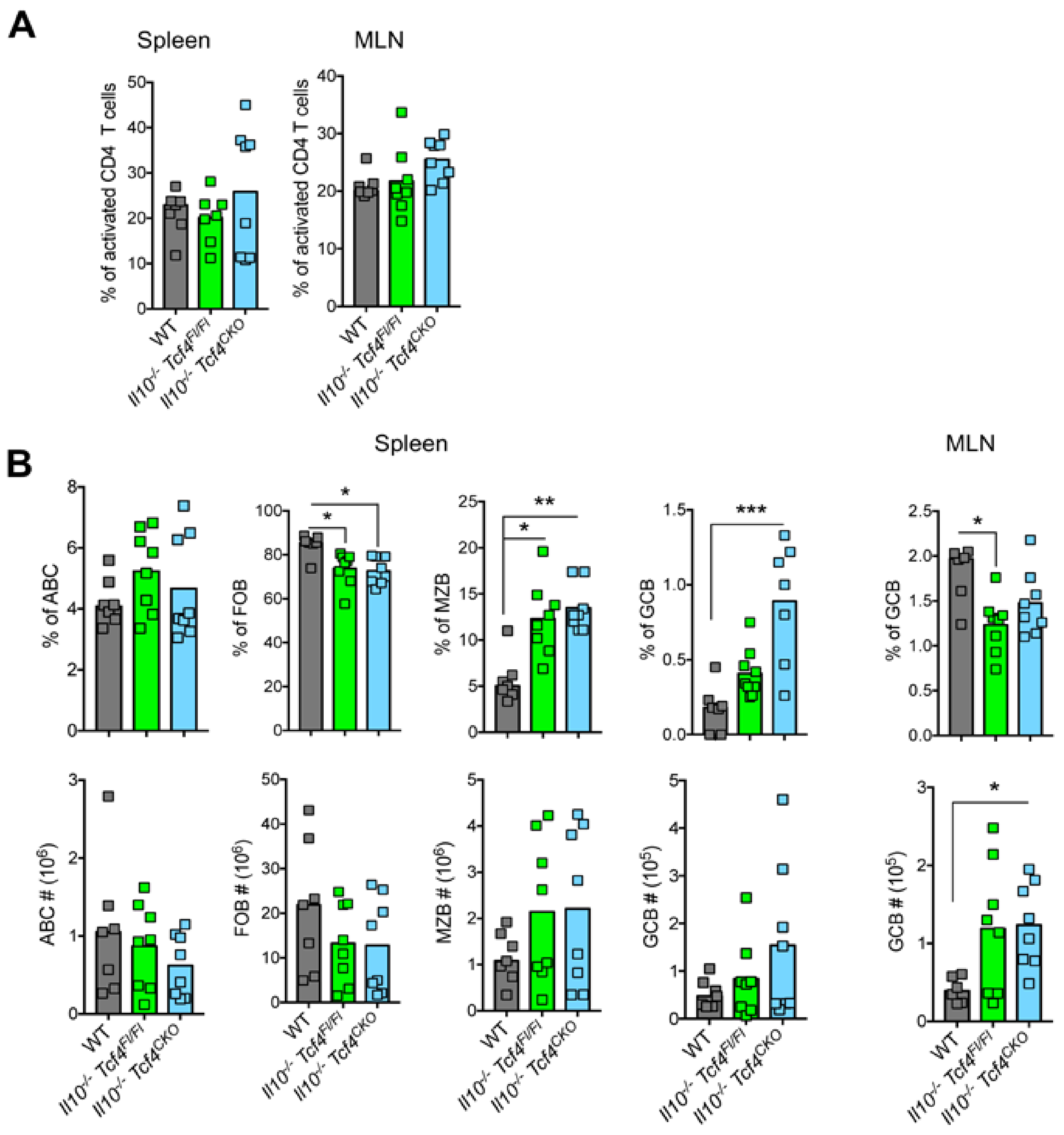


**FigS6.** DC-specific deletion of *Tcf4* does not impact the overall immune activation in *Il10*-deficient mice

| Target | Fluorochrome | Clone | Dilutions | Source | Reference |
|---|---|---|---|---|---|
| Sca1 (Ly-6A/A) | FITC | D7 | 1/200 | Thermo Fisher | 11-5981-82 |
| CD86 (B7-2) | FITC | GL1 | 1/200 | Thermo Fisher | 11-0862-82 |
| CD69 | FITC | H1.2F3 | 1/200 | Thermo Fisher | 11-0691-82 |
| PD1 (CD279) | FITC | RMP1-30 | 1/100 | Thermo Fisher | 11-9981-82 |
| Streptvidin | FITC | | 1/200 | Thermo Fisher | 11-4317-87 |
| CD45 RB | PE | C363.16A | 1/400 | Thermo Fisher | 12-0455-82 |
| CD11c | PE | N418 | 1/400 | Thermo Fisher | 12-0114-82 |
| CD21/35 | PE | eBio4E3 | 1/200 | Thermo Fisher | 12-0212-82 |
| FoxP3 | PE | FJK-16s | 1/100 | Thermo Fisher | 12-5773-82 |
| CD95 (Fas) | PE | 15A7 | 1/200 | Thermo Fisher | 12-0951-81 |
| BCL6 | PE | K112-91 | 1/100 | BD-Bioscience | 561522 |
| CD44 | APC | IM7 | 1/400 | Thermo Fisher | 17-0441-82 |
| Siglec H | APC | 551 | 1/200 | Biolegend | 129612 |
| AA4.1 (CD93) | APC | AA4.1 | 1/200 | Thermo Fisher | 17-5892-82 |
| CD25 | APC | PC61.5 | 1/200 | Thermo Fisher | 17-0251-82 |
| CD83 | APC | Michel-19 | 1/200 | Biolegend | 121509 |
| Streptavidin | APC | | 1/400 | Thermo Fisher | 45-4317-80 |
| TCRb | APC-eFluor 780 | H57-597 | 1/150 | Thermo Fisher | 47-5961-82 |
| CD11b | APC-eFluor 780 | M1/70 | 1/400 | Thermo Fisher | 47-0112-82 |
| CD4 | PE-Cy7 | GK1.5 | 1/800 | Thermo Fisher | 25-0041-82 |
| CD8a | PE-Cy7 | 53-6.7 | 1/800 | Thermo Fisher | 25-0081-82 |
| CD23 | PE-Cy7 | B3B4 | 1/400 | Thermo Fisher | 25-0232-82 |
| B220 (CD45R) | PE-Cy7 | RA3-6B2 | 1/800 | Thermo Fisher | 25-0452-82 |
| B220 (CD45R) | eFluor 450 | RA3-6B2 | 1/200 | Thermo Fisher | 48-0452-82 |
| MHC II (I-A/I-E) | eFluor 450 | M5/114.15.2 | 1/400 | Thermo Fisher | 48-5321-82 |
| CD8a | Percp Cy5.5 | 53-6.7 | 1/800 | Thermo Fisher | 45-0081-82 |
| CXCR3 (CD183) | Percp Cy5.5 | CXCR3-173 | 1/200 | Thermo Fisher | 45-1831-82 |
| Streptavidin | Percp Cy5.5 | | 1/400 | Thermo Fisher | 17-4317-82 |
| CD40 | Biotin | 1C10 | 1/200 | Thermo Fisher | 13-0401-82 |
| Peanut Agglutinin | Biotin | | 1/1000 | Vector labs | B-1075 |
| CXCR5 (CD185) | Biotin | SPRCL5 | 1/100 | Thermo Fisher | 13-7185-82 |

**Table S1.** List of antibodies used in this study.
